# Endoscopic-Assisted Excision of Frontal Bone Osteomas: A Case Series Study of Technical Considerations and Complications from a Single Surgeon

**DOI:** 10.3390/medicina62050947

**Published:** 2026-05-13

**Authors:** Gabrielle Odoom, Lamorna Coyle, Ashley Howell, Sydney Barone, Neil Tanna

**Affiliations:** 1 Division of Plastic and Reconstructive Surgery, Northwell Health, Great Neck, NY 11021, USA; godoom@northwell.edu (G.O.); sbarone1@northwell.edu (S.B.); 2 Donald & Barbara Zucker School of Medicine at Hofstra/Northwell, Hempstead, NY 11021, USA; lcoyle1@pride.hofstra.edu (L.C.); ahowell4@northwell.edu (A.H.)

**Keywords:** osteoma, endoscopy, retrospective studies, bone and bones, minimally invasive surgical procedures, frontal bone

## Abstract

*Background and Objectives*: Frontal bone osteomas are benign tumors characterized by the abnormal proliferation of bone. Though typically asymptomatic, these masses often produce visible forehead deformity and may cause localized discomfort. Endoscopic resection has emerged as an optimal technique for appropriately selected frontal osteomas because it enables complete excision while minimizing visible scarring and risk of neurovascular injury. This case series reviews the senior author’s patient outcomes and complications using a scalp-based, endoscopic-assisted surgical approach for frontal bone osteoma excision. *Materials and Methods*: A review was conducted of all patients (*n* = 22) who underwent endoscopic-assisted frontal bone osteoma excision performed by a single surgeon between February 2019 and December 2025. All procedures involved endoscopic visualization through remote scalp incisions, osteoma excision, and frontal bone contouring. Patient demographics, presenting symptoms, CT imaging findings, operative details, histopathology results, and postoperative outcomes were recorded. *Results*: Twenty-two patients underwent endoscopic-assisted excision of frontal bone osteomas performed by the senior author (NT). The primary concern prompting surgical intervention was aesthetic deformity, with localized pain reported in some cases. For all patients, CT imaging was obtained preoperatively to evaluate lesion size and anatomical relationships to nearby facial structures. Histopathology confirmed cortical bone consistent with osteoma in all cases. Postoperative swelling and ecchymosis were common in the first week but resolved spontaneously. No contour irregularities, fluid collections, residual bone, or sensory deficits were observed. Some alopecia was noted in the first three patients, prompting the senior author to modify the technique. All patients healed with small, flat, well-concealed scars adjacent to the hairline. *Conclusions*: This single-surgeon experience demonstrates that endoscopic-assisted excision of frontal bone osteomas is a safe and effective technique that permits complete excision with excellent aesthetic outcomes and minimal morbidity. The operative framework presented in this study, including surgical planning, technique steps, and postoperative management, supports endoscopic resection as an ideal alternative to traditional direct approaches for frontal bone osteomas.

## 1. Introduction

Osteomas are benign osteogenic tumors characterized by the slow, continuous proliferation of mature bone [1,2]. These lesions occur most frequently in the skull and represent the most common benign tumor observed in the craniofacial skeleton, with reported prevalence ranging from 0.014% to 0.43% across systematic reviews and case series [3,4,5,6]. The true incidence of osteomas is difficult to ascertain, as most are asymptomatic and discovered incidentally through visible anatomic deformity or radiologic imaging. Osteomas present most often in early to mid-adulthood with a slight male predilection variably reported in the literature [7,8]. Though the precise etiology of osteomas remains unclear, proposed contributing factors include local trauma, genetic mutations, and hereditary disorders such as Gardner’s syndrome [4,9].

Histologically, osteomas can be categorized into compact and cancellous subtypes, with mixed types also reported [5]. Compact osteomas consist of dense cortical bone, while cancellous osteomas consist of spongy bone with interspersed marrow spaces. Osteomas of all histological subtypes are hypothesized to have a characteristic zonal structure with a vascularized and metabolically active core surrounded by a less active peripheral region [7]. This hypothesized structure matches the centrifugal growth pattern often observed in osteomas, suggesting that excision of the core may successfully halt the lesion growth.

Craniofacial osteomas can emanate from the skull periosteum or endosteum, paranasal sinuses, maxilla, or mandible [10]. Frontal bone osteomas emerge from the periosteum and present as firm, immobile masses that are distinguishable from the surrounding contour [3]. While most frontal bone osteomas are asymptomatic, they can trigger headaches or localized discomfort in some cases [7,10]. The primary indication for surgical intervention is aesthetic deformity, as these osteomas typically produce visible forehead contour irregularities.

Traditionally, direct approaches have been used for frontal bone osteoma removal. However, while direct excision provides reliable lesion exposure, it often results in conspicuous forehead scars and can require extensive soft tissue dissection that may disrupt neurovascular structure and sensation [7]. Since its initial description by Onishi et al. in 1995, the endoscopic-assisted approach has emerged as an increasingly favored alternative for appropriately selected frontal bone osteomas [11]. By utilizing incisions concealed in the hairline for precise endoscopic visualization, this technique offers optimal aesthetic outcomes with minimal neurovascular morbidity [4,7,11,12].

Despite the growing adoption of the endoscopic-assisted technique for frontal bone osteoma management, there is a paucity of large single-surgeon series on this method. By minimizing technique variability, single-surgeon series importantly allow for more consistent outcome assessments [13,14,15]. The present case series reports the procedural indications, operative technique, perioperative management, and clinical outcomes from a single surgeon’s experience performing endoscopic-assisted excisions of frontal bone osteomas. This study aims to define the safety, efficacy, and aesthetic outcomes of this minimally invasive approach through a case series evaluation.

## 2. Materials and Methods

All patients who underwent endoscopic-assisted frontal bone osteoma excision performed by the senior author (NT) from February 2019 to December 2025 were included in this retrospective analysis. This study was conducted according to PROCESS guidelines and deemed exempt for approval by the Institutional Review Board. Deidentified demographic data from 22 patients were analyzed, including age, sex, and body mass index (BMI). Clinical and operative details for each case were also recorded. Outcome variables including length of follow-up, adverse postoperative events, complication management, and pathology results were assessed. The sample size evaluated reflects the patient population available during the study period and was not statistically powered for a comparable analysis. All 22 patients underwent the same technique of endoscopic-assisted frontal bone osteoma excision as described below. Prior to surgery, all patients provided written informed consent after a detailed discussion about the risks, potential complications, and the possibility of converting to an open approach if needed.

### 2.1. Preoperative Marking and Operative Setup

**Figure 1:**
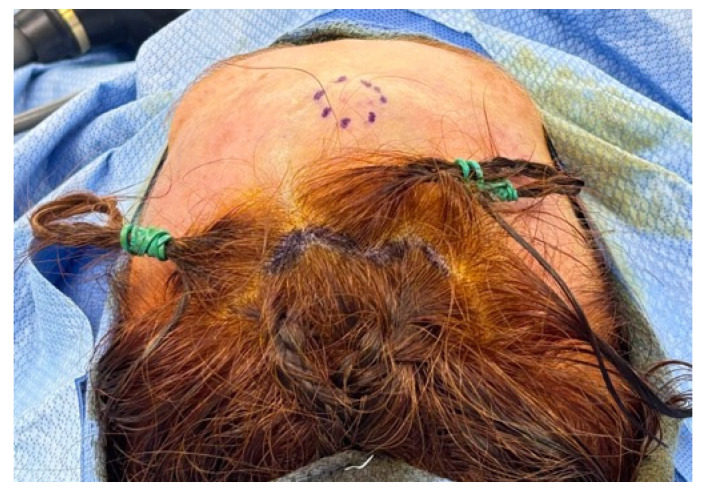
Preoperative markings are shown.

The boundaries of the osteoma are marked on the forehead preoperatively with the patient in an upright position. No hair is cut or shaved for surgery. Before initiating dissection, intravenous prophylactic antibiotics and deep vein thrombosis prophylaxis are administered according to institutional protocols. The patient is placed in a supine position under general anesthesia with all points subject to pressure appropriately padded (Figure 1).

### 2.2. Incision and Initial Dissection

A zigzag incision of 3–4 cm in linear length is made about two centimeters posterior to the hairline with a #15 blade scalpel (Video S1).

Initial dissection is performed through the scalp skin, subcutaneous tissue, through the periosteum, and down to the calvarium. Then, subperiosteal dissection is performed with a Freer or Cottle elevator in a posterior-to-anterior direction to expose the frontal bone (Video S2).

### 2.3. Endoscopic-Assisted Dissection and Removal

Once the frontal bone is adequately exposed, dissection transitions to the endoscopic-assisted approach using a zero-degree, 4 mm endoscope. Using endoscopic visualization, the anterior forehead flap is elevated off the frontal bone until the osteoma is encountered (Video S3).

**Figure 2:**
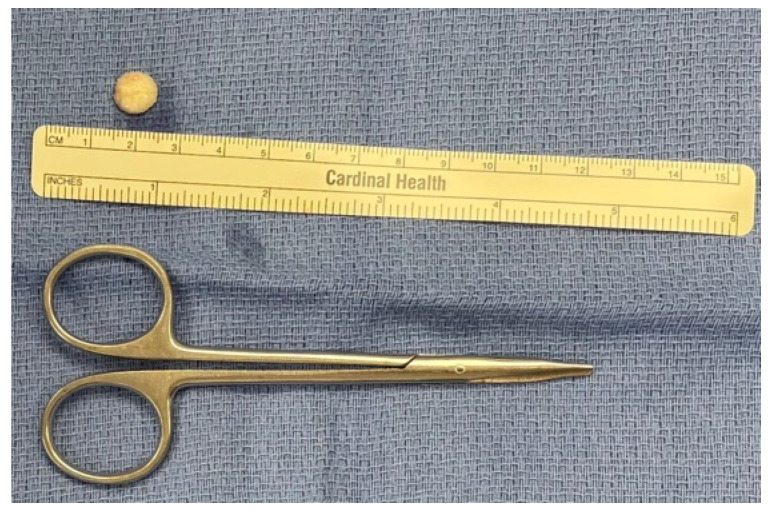
Osteoma specimen is sent for histopathology.

The osteoma is excised with a 2 mm osteotome and submitted to pathology for histological confirmation (Videos S4 and S5, Figure 2).

After excision, frontal bone contouring is performed using either a rasp or diamond burr to restore a natural and smooth forehead contour (Video S6). Any remaining bone fragments and chips are cleared from the surgical field using copious irrigation.

### 2.4. Wound Closure

The scalp incision is closed in layers (Video S7). Deep dermal closure is performed with 3-0 Monocryl™ sutures (Ethicon, Raritan, NJ, USA) in interrupted buried fashion. The superficial layer is closed with non-absorbable 5-0 polypropylene (PROLENE™) (Ethicon, Raritan, NJ, USA) sutures placed in simple interrupted fashion. The incision site is cleaned, and bacitracin ointment is applied to the suture line.

### 2.5. Compression Dressing

A compression dressing is placed on the patient’s forehead to reduce postoperative edema and ecchymosis. Gauze is positioned over the forehead and hairline incision, secured with a Kerlix^®^ bandage (Medtronic, Dublin, Ireland), and then reinforced with an Ace bandage wrap that is wrapped vertically (from chin to ears and top of head) and horizontally (circumferentially around the forehead) to provide uniform compression. The patient is instructed to keep this dressing in place for 1–2 days postoperatively.

### 2.6. Postoperative Management

Patients were seen postoperatively within 7 to 14 days. At the first postoperative visit, the nonabsorbable sutures are removed, the incision is assessed for healing and signs of complications, and the patient’s symptoms are evaluated. Additional follow-up visits are conducted on an as-needed basis.

## 3. Results

### 3.1. Patient Characteristics & Clinical Presentations

During the study period, a total of 22 patients underwent endoscopic-assisted frontal bone osteoma removal with the senior author (NT). The cohort included 21 females and 1 male. All patients presented with a firm, immobile forehead mass that was free from the overlying skin, measuring approximately 0.5–2.0 cm on examination and consistent with an osteoma. In 20 patients, the mass was present for several years before presentation with gradual growth over time. Two patients incidentally discovered the mass recently before clinical evaluation.

Medical history was unremarkable in most patients (21/22), with 1 patient reporting history of blunt trauma to the forehead region. Fourteen patients were asymptomatic, while 8 reported symptoms including local tenderness, headaches, or migraines. In all cases, visible deformity of the forehead contour was the primary concern prompting patients to pursue surgical intervention. There were no cases of drainage with palpation, overlying skin changes, or sensory disturbances observed on clinical examination.

The mean age of the cohort was 45 years (range 20–56 years), and the mean body mass index (BMI) was 26.1 kg/m^2^. For all patients, procedural indications were confirmed preoperatively with a computed tomography (CT) scan of the head to assess osteoma size and anatomical relationship to adjacent structures. Imaging results for all patients showed well-defined, dense masses emanating from the frontal bone, consistent with osteoma.

### 3.2. Specimen Sizes and Pathology Reports

All excised specimens were measured intraoperatively. Consistent with the estimates from preoperative evaluation and imaging, the mean osteoma diameter was 1.3 cm (size range 0.6–2 cm). Histopathology results indicated benign mature bone that was consistent with osteoma across all cases. No specimens exhibited atypia or malignant features.

### 3.3. Complications and Postoperative Courses

All procedures were performed in an outpatient setting at a single institution by the senior author. Postoperative complications occurred in 3 patients, all of whom experienced mild, localized alopecia at the incision site. There were no additional complications, such a seroma, hematoma, infection, sensory nerve damage, or contour irregularities. No drains were used, and no patients had delayed recovery after surgery.

The mean follow-up duration was six weeks, with an average of two postoperative visits per patient. Prior to the three cases of postoperative alopecia, the routine follow-up protocol consisted of two visits, including an early evaluation followed by a long-term evaluation to discuss scar care. After the three occurrences of alopecia, the senior author modified the incision closure technique to use a greater proportion of nonabsorbable sutures and less absorbable sutures to reduce scalp inflammation and mitigate the risk of hair loss. This modification necessitated an additional early postoperative visit within the first two weeks for suture removal. From thereon, long-term follow-up was conducted on an as-needed basis.

**Figure 3:**
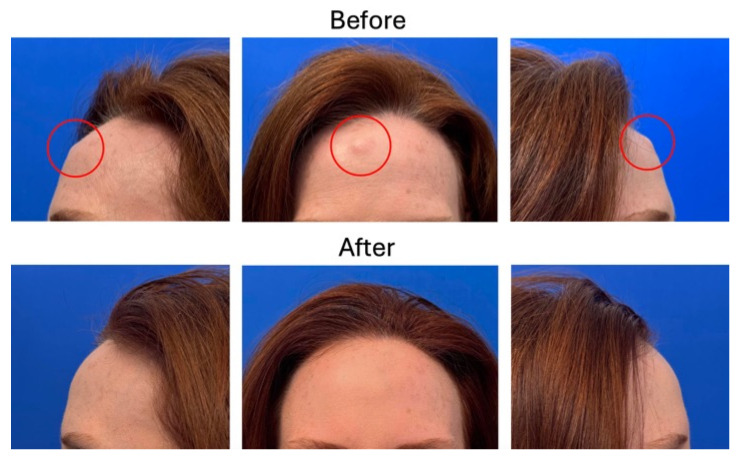
Preoperative and postoperative results for a patient are shown in frontal and bilateral views.

At their final follow-up visits, no patients presented with residual bony prominence or contour irregularity of the forehead. All incisions healed nicely as flat and narrow scars concealed in the hairline (Figure 3). There was high patient satisfaction with the aesthetic outcome of the procedure across the cohort. Additionally, patients who presented with headaches and tenderness reported symptomatic improvement. No cases of osteoma recurrence were identified in the follow-up period.

## 4. Discussion

Management of frontal bone osteomas has evolved significantly over the past three decades. Direct excision though an open forehead incision was once considered the gold standard, however, the advent of the endoscopic approach in the 1990s revolutionized the surgical management of osteomas (Video S8) [11,16]. Many surgeons have since adopted this minimally invasive technique for both its superior aesthetic outcomes and reduced patient morbidity. Compared with the traditional direct approach, endoscopic excision of forehead osteomas is associated with reduced risks of hematoma and infection, as well as favorable scarring that is well-concealed by the hairline [7,17,18]. Improved scarring is particularly advantageous given that aesthetic concerns are the primary reason why patients seek surgical intervention for these lesions.

Most forehead osteomas remain asymptomatic and are discovered incidentally when they create visible facial profile irregularities. Other less common indications may include localized pain due to nerve compression, as well as osteomas demonstrating rapid growth [19]. In this series, visible contour deformity was the universal presenting concern prompting patients to pursue consultation and subsequent surgical intervention.

Forehead osteoma excision is performed by both plastic surgeons and otolaryngologists, with otolaryngologists more commonly involved when the osteoma extends into the sinonasal region or has complex anatomical connections to critical facial structures. In both fields, endoscopic-assisted techniques have become standard practice for a wide array of procedures, including osteoma management. Within plastic and reconstructive surgery, endoscopic approaches have demonstrated success in achieving inconspicuous scarring in many cosmetic procedures, including abdominoplasty, breast augmentation, and facial rejuvenation [20]. Endoscopic techniques have also proven effective in minimizing surgical morbidity in free and pedicled flap harvests, tissue expander placement, and carpal tunnel release [21,22,23,24,25]. The success of the endoscopic approach across this diverse spectrum of procedures supports its application to frontal bone osteoma removal, where both aesthetic outcome and reduced morbidity are key goals for achieving patient safety and satisfaction.

The traditional direct surgical technique employed for forehead osteoma removal involves a visible incision along the forehead. Though this method produces excellent tumor visibility and access, it inevitably produces a prominent facial scar in patients who frequently otherwise have an asymptomatic course [26]. Thus, a primary benefit of endoscopic approaches to forehead osteoma removal has been the ease of hiding resulting scars, as it requires a smaller incision that is well-concealed within the hair-bearing scalp [12]. Specifically, a zigzag incision is chosen due to its ease of concealability within the hairline. As the incision line itself is likely to experience a degree of alopecia, a zigzag formation offers the ability to cover satellite areas of hair loss through innovative hair parting tactics. Additionally, the zigzag reduces tension on the incision line, further mitigating both scar formation and the associated extent of alopecia.

Frontal bone endoscopy faces a unique technical challenge that necessitates a hybrid surgical approach. Unlike other endoscopic surgical approaches, such as arthroscopy and endoscopic sinus surgery, which exploit a naturally existing potential space for access and visibility, there exists no natural cavity within the forehead that can be directly used for endoscopic instrumentation [27,28]. It is therefore necessary for the surgeon to create an artificial anatomic space in which the endoscope may be manipulated. The choice to begin the procedure using an open technique in the scalp provides the surgeon with greater access and maneuverability when creating this potential space. In this initial open phase, the surgeon can precisely control the borders of the dissection pocket to minimize the risks of bleeding and infection [26]. A subsequent transition to endoscopy can occur after the appropriate surgical plane has been visualized, and sufficient elevation of the periosteum has been achieved.

The zero-degree, 4 mm endoscope offers excellent visibility of the osteoma, equipped with high-definition imaging power and an ergonomic handle to prevent surgeon fatigue during dissection. In addition to its primary surgical function, the endoscope allows for intraoperative photographic documentation that can be valuable for teaching purposes or future surgical planning. Tumor resection proceeds with an osteotome, often with the assistance of a mallet. By positioning the osteotome superiorly, the bony lesion can be removed en bloc or chipped away in piecemeal fashion while continually traveling in an inferior direction. Copious irrigation facilitates the removal of bone fragments, which is essential to prevent infection, recurrence, or the subsequent development of other contour deformities caused by residual shards. Once removed, the base of the lesion can be contoured with a diamond burr or rasp to prevent any residual contour deformities and provide a smooth osseous foundation.

Wound closure is accomplished with a layered technique using both absorbable and nonabsorbable sutures. Deep dermal approximation is performed using 3-0 Monocryl™ sutures. The senior author (NT) transitioned to using less absorbable sutures and more nonabsorbable monofilament sutures after observing postoperative alopecia in a patient whose incision has been closed entirely with absorbable sutures. Nonabsorbable sutures such as polypropylene or nylon are now utilized for cutaneous closure, as these sutures incur minimal inflammation to surrounding tissue, making them ideal for optimizing scar quality and mitigating alopecia. After this technique modification, no additional cases of alopecia have been observed.

Dressing choice is also key to successful operative outcomes. Compression dressings are left in place for 24 to 48 h to prevent the formation of potential spaces, limiting the risk of ecchymosis and swelling. Early compression also helps control scarring by mitigating hypertrophic or keloid scar formation at or beyond the incision [29]. Finally, appropriate dressings may help prevent hair entrapment within the healing surgical site, which may present an opportunity for infection and related complications.

### Limitations

Though the endoscopic approach to osteoma excision provides optimal results in most patient populations, the open technique may be more appropriate for some demographics. Male patterned baldness necessitates particular attention, as a posteriorly displaced hairline may convolute incision placement. The endoscopic approach remains valuable in most men with a sparse or receding hairline, though excessive posterior displacement may inhibit access to the surgical site. In such instances, a traditional open approach may be necessary to achieve the desired aesthetic outcome, or alternatively, placing the endoscope through a strategic incision embedded within a natural forehead crease may offer optimal cosmesis [30].

This case series contributes important insight into the technique of endoscopic osteoma removal through the lens of a single provider, providing outcomes data on patients who have undergone the same procedure. While this limits the number of patients that may be included, this report still offers one of the largest cohorts of a single surgeon study on this topic to date. Future studies should aim to collect additional data on patients undergoing endoscopic osteoma resection with the operating surgeon (NT) to strengthen the analysis and expand the generalizability of the findings. In addition, future work should utilize a longer follow-up period to ensure that recurrence rates are accurately captured, particularly given that osteomas are slow-growing tumors. While this study does not establish a strict size limit for the endoscopic-assisted approach, the maximum osteoma diameter in our cohort was 2.0 cm. Conclusions regarding the feasibility of the endoscopic-approach for larger osteomas cannot be drawn. Future studies including lesions exceeding this size are warranted to determine the appropriate size threshold for this approach.

It is important to note that this single-surgeon case series is subject to inherent sources of bias. There is a risk of selection bias given that this study only included patients who underwent surgery, with those who presented with frontal bone osteomas and declined surgery or sought care elsewhere not represented. Performance bias is also inherent to a single-surgeon study design, as outcomes reflect the technique and experience of one surgeon and may not be generalizable to other providers. Although this study is retrospective in design, all patient charts were complete with no missing data, mitigating concerns related to retrospective data collection bias.

## 5. Conclusions

This single-surgeon series presents a reproducible technique for endoscopic-assisted excision of frontal bone osteomas alongside a consistent evaluation of patient outcomes. The cumulative experience from this single-surgeon cohort supports the endoscopic approach as a safe, effective, and cosmetically favorable alternative to traditional direct excision. Across all cases, endoscopic-assisted visualization and dissection through a remote scalp incision enabled complete lesion excision, with minimal morbidity and no recurrences during the follow-up period. Notably, the three occurrences of postoperative alopecia led to modification of the closure technique by increasing the proportion of nonabsorbable sutures, after which no further cases of hair loss occurred. This finding, along with other technical pearls related to the incision design and postoperative compression dressings, enhances the practical value of the operative framework presented. The inclusion of intraoperative images and video-based demonstrations of the technique further supports reproducibility and adoption by other surgeons. Overall, the findings support endoscopic-assisted frontal bone osteoma excision as an ideal technique for obtaining excellent aesthetic outcomes while maintaining safety and efficacy.

## Data Availability

The original contributions presented in this study are included in the article/Supplementary Materials. Further inquiries can be directed to the corresponding author.

## Supplementary Materials

The following supporting information can be downloaded at: https://www.mdpi.com/article/10.3390/medicina62050947/s1, Video S1: Surgical Incision Made. Video S2: Exposure of Frontal Bone with Direct Dissection. Video S3: Endoscopic Dissection Encounters Osteoma. Video S4: Excision with Osteotome and Mallet. Video S5: Removal of Specimen from Field. Video S6: Frontal Bone Contouring. Video S7: Deep Dermal and Superficial Closure. Video S8: Comparison of Direct vs Endoscopic Approach.
